# Supplementary material for: An image-based flow cytometric approach to the assessment of the nucleus-to-cytoplasm ratio
Source: PLoS One. 2021 Jun 24;16(6):e0253439. doi: 10.1371/journal.pone.0253439 (PMC8224973; doi:10.1371/journal.pone.0253439)
Supplement: S1 Table — (DOCX) [file pone.0253439.s001.docx]

**Sebastian et al., “An image-based flow cytometric approach to the nucleus-to-cytoplasmic ratio” Supplementary Table 1:**

**S1 Table** - A summary of IFC measurements and corresponding standard deviations for the cell and nucleus diameter as well as the N:C ratio for each cell line using the modified IFC workflow on all non-malignant and malignant cell lines.

| **Cell Line** | OCI-AML-5 (n=4510) | CAKI-2 (n=799) | HT-29 (n=5309) | SK-BR-3 (n=1596) | MCF-10A (n=1109) |
| --- | --- | --- | --- | --- | --- |
| **Cell Diameter [μm]** | 12.2 ± 1.1 | 24.3 ± 2.3 | 16.0 ± 1.7 | 17.2 ± 3.4 | 19.4 ± 2.2 |
| **Nucleus Diameter [μm]** | 8.8 ± 1.0 | 15.0 ± 1.7 | 11.0 ± 1.2 | 11.7 ± 1.9 | 10.1 ± 1.8 |
| **N:C** | 0.73 ± 0.07 | 0.62 ± 0.06 | 0.69 ± 0.07 | 0.70 ± 0.13 | 0.53 ± 0.11 |
