# Supplementary material for: An image-based flow cytometric approach to the assessment of the nucleus-to-cytoplasm ratio
Source: PLoS One. 2021 Jun 24;16(6):e0253439. doi: 10.1371/journal.pone.0253439 (PMC8224973; doi:10.1371/journal.pone.0253439)
Supplement: Attachment — Submitted filename: Sebastian_et_al_Rebuttals_SubmissionCopy.docx [file pone.0253439.s002.docx]

**Sebastian et al. PLOS ONE Reviewer Rebuttals**

We thank the editors and reviewers for thoughtfully reading our study and providing feedback, especially during these stressful times. The references and reference numbering provided in the replies to each Reviewer comment corresponds to a reference from the re-submitted manuscript.

************************************

Reviewer #1:

1. The manuscript reports a method for assessing N:C ratios in 4 cancer cell lines using DRAQ5 as a nuclear stain and imaging flow cytometry. The cell lines are exemplars to illustrate the approach. In addition, the N:C ratio needs to be compared with normal cells. This is especially relevant if the intent is to use this strategy to detect CTC; in this situation data of N:C for normal nucleated cells in blood (i.e. leucocytes) should be assessed.

This is an excellent suggestion. Our revised manuscript now incudes N:C data using the MCF10A cell line (a known normal breast cell model derived from non-malignant breast tissue). Results from these non-malignant cell measurements have been included in Table 1 (Page 11, Line 205-207) and Fig 2 (Page 12, Line 208-210), and are provided below for convenience.

**Table 1** - A summary of IFC measurements and corresponding standard deviations for the cell and nucleus diameter as well as the N:C ratio for each cell line.

| **Cell Line** | OCI-AML-5 (n=5284) | CAKI-2 (n=1096) | HT-29 (n=6302) | SK-BR-3 (n=3159) | MCF-10A (n=1109) |
| --- | --- | --- | --- | --- | --- |
| **Cell Diameter [μm]** | 12.3 ± 1.2 | 24.5 ± 2.6 | 16.2 ± 1.8 | 18.0 ± 3.7 | 19.4 ± 2.2 |
| **Nucleus Diameter [μm]** | 9.0 ± 1.1 | 15.6 ± 2.1 | 11.2 ± 1.3 | 12.5 ± 2.1 | 10.1 ± 1.8 |
| **N:C** | 0.73 ± 0.07 | 0.64 ± 0.08 | 0.69 ± 0.07 | 0.71 ± 0.13 | 0.53 ± 0.11 |

**Fig 2. Cell diameter, Nuclear diameter, and N:C ratio distribution.** (a, c, e) Beeswarm plots and (b, d, f) density distributions display (a, b) cell, (c, d) nucleus, and (e, f) N:C ratio distribution across cell lines. Triple asterisks indicate a *p*-value of <0.001.

To obtain accurate N:C measurements, we modified our analysis template for the MCF-10A cells. Initially when calculating the N:C ratio using the **same analysis template as the malignant cell lines**, we calculated the following results:

**Table 2** - A summary of IFC measurements and corresponding standard deviations for the cell and nucleus diameter as well as the N:C ratio for each cell line using the malignant IFC workflow.

| **Cell Line** | OCI-AML-5 (n=5284) | CAKI-2 (n=1096) | HT-29 (n=6302) | SK-BR-3 (n=3159) | MCF-10A (n=21153) |
| --- | --- | --- | --- | --- | --- |
| **Cell Diameter [μm]** | 12.3 ± 1.2 | 24.5 ± 2.6 | 16.2 ± 1.8 | 18.0 ± 3.7 | 19.4 ± 2.4 |
| **Nucleus Diameter [μm]** | 9.0 ± 1.1 | 15.6 ± 2.1 | 11.2 ± 1.3 | 12.5 ± 2.1 | 12.8 ± 2.0 |
| **N:C** | 0.73 ± 0.07 | 0.64 ± 0.08 | 0.69 ± 0.07 | 0.71 ± 0.13 | 0.67 ± 0.13 |

When comparing Table 1 and Table 2, note that the cell diameters are the same, the nucleus diameters differ, and we went from 21153 measurements to 1109.

When examining the IFC images of the MCF-10As, we observed noticeable dye leakage in the IFC images of the MCF-10As (unseen in other cell lines to this level of severity). The culturing process for the MCF-10As requires a long immersion time in trypsin (up to 20 minutes). It has been previously published that the **extended immersion** in the trypsin solution could cause damage to the cellular membrane (1), proteomic alterations (2), decrease in cellular protein expression (3), and damage to nuclear proteins (4). We hypothesize that the long immersion time in trypsin may have cause the DRAQ-5 to leak out of nucleus. This leakage would cause the IFC post-processing workflow to incorrectly overestimate the nuclear diameters of the MCF-10A cells. As per the suggestion of Reviewer 1’s fifth comment, we added a single extra gate to our post-processing workflow to including a gate to focus on cells with high gradient RMS values in their fluorescent images to account for this effect.

Thus, Table 1 only applies this single extra gate to the MCF-10A cells since these cells were the only ones with severe dye leakage. We applied this modified template (e.g., with the single extra gate) on all four malignant cell lines and saw minimal change to the N:C ratios (shown in Table 3). The main change in Table 3 was the lower sample population analyzed; however, with an extra gate added to the IFC workflow to exclude out-of-focus fluorescent images, this is to be expected.

**Table 3** - A summary of IFC measurements and corresponding standard deviations for the cell and nucleus diameter as well as the N:C ratio for each cell line using the modified IFC workflow on all non-malignant and malignant cell lines.

| **Cell Line** | OCI-AML-5 (n=4510) | CAKI-2 (n=799) | HT-29 (n=5309) | SK-BR-3 (n=1596) | MCF-10A (n=1109) |
| --- | --- | --- | --- | --- | --- |
| **Cell Diameter [μm]** | 12.2 ± 1.1 | 24.3 ± 2.3 | 16.0 ± 1.7 | 17.2 ± 3.4 | 19.4 ± 2.2 |
| **Nucleus Diameter [μm]** | 8.8 ± 1.0 | 15.0 ± 1.7 | 11.0 ± 1.2 | 11.7 ± 1.9 | 10.1 ± 1.8 |
| **N:C** | 0.73 ± 0.07 | 0.62 ± 0.06 | 0.69 ± 0.07 | 0.70 ± 0.13 | 0.53 ± 0.11 |

Thus, the new manuscript will present the original template applied to all four malignant cell lines and the extra gate applied to the MCF-10A cells with Table 3 provided as Supplementary Table 1 and with the rationale explained in the manuscript.

The subjectivity and limitations of thresholding were included in the limitations portion of our Discussion section (in addition to many other mentioned limitations of the technique) as follows (Page 14, Line 309-317):

Initially, using the malignant cell line workflow, we noticed DRAQ-5 leakage out of the nucleus in the analyzed IFC images. We hypothesize that the extended immersion in the trypsin solution could have caused damage to the cell membrane (43), proteomic alterations (44), decrease in cellular protein expression (45), and damage to the nuclear proteins (46). To account for this leakage, we added an extra gradient-root-mean-squared gate to isolate fluorescent cell images with sharp nuclear boundaries that were highly focused. This modified template produced minimal changes to the N:C ratio when applied on all four malignant cell lines (see S1 Table) but did lower the sample population that was analyzed. This subjective gating and masking approach is a limitation to imaging flow cytometry.

In addition, we added information to our Methods section to describe the approach used (Page 7, Line 141-145):

DRAQ-5 leakage out of the nucleus was addressed in our workflow for the non-malignant cells by adding a plot that used gradient root-mean-squared feature on the fluorescent images to isolate cells that had localization of the DRAQ-5 dye in their nucleus (high gradient RMS value). This plot is provided in Fig 1b). We applied this extra gate on all cell lines and saw minimal changes in the N:C ratio across the cancer cell lines (see S1 Table).

Moreover, the additional gradient root-mean-squared plot on the fluorescent images is included in our modified Fig 1:

**Fig 1. IFC analysis workflow and representative cells.** (a) The IFC analysis workflow excludes: (I) unfocused cells, (II) multiple cells per brightfield image, (III), clipped cells in brightfield images, (IV) unstained cells. Sequential gating bars (I - blue, II - green, III - yellow, IV - orange) depict manually gated ROIs for each cell population. (b) Additional gating step for MCF-10A cells to address leakage due to extended trypsinization. (c-g) Representative cells from each cell line after IFC gating cell mask (left column), stained fluorescent image (middle column), and cytoplasm (right column).

43. Revel JP, Hoch P, Ho D. Adhesion of culture cells to their substratum. Experimental Cell Research. 1974 Mar;84(1–2):207–18.

44. Huang H-L, Hsing H-W, Lai T-C, Chen Y-W, Lee T-R, Chan H-T, et al. Trypsin-induced proteome alteration during cell subculture in mammalian cells. J Biomed Sci. 2010;17(1):36.

45. Gräbner R, Till U, Heller R. Flow cytometric determination of E-selectin, vascular cell adhesion molecule-1, and intercellular cell adhesion molecule-1 in formaldehyde-fixed endothelial cell monolayers. Cytometry. 2000 Jul 1;40(3):238–44.

46. Maizel A, Nicolini C, Baserga R. Effect of cell trypsinization on nuclear proteins of WI-38 fibroblasts in culture. J Cell Physiol. 1975 Aug;86(1):71–81.

2. If this work is merely to describe the method, then the precise biology of each cell line is not relevant. This comment is made as the details of the cell lines are not provided. Are any of the cell lines aneuploidy? Of particular note is the “AML cell” line which has no name or details. There are many subtypes of acute myeloid leukaemia and they can be heterogeneous with varying nuclear size, shape and complexity by subjective appearance by light microscopy. These cells are likely to have a broad range of N:C ratios.

Our revised manuscript now includes the name of the exact strain of the AML cell line chosen (OCI-AML-5). In the “Methods” section, the description of the company used for acquisition of the cell line and the corresponding catalogue number was included in the revised manuscript in the “Methods” section (Page 5 Line 96**)** as: OCI-AML-5 cells (DSMZ, Braunschweig, Germany, ACC247). .

In response to the Reviewer’s first sentence of this comment, **the aim of this work is to describe a novel high-throughput method for assessing the N:C ratio of various cell lines**. It is beyond the current abilities of this analysis method to determine the anatomical site of origin of a given cell based on the IFC derived characteristics. We have made this limitation more explicit as in the “Discussion” section of the manuscript as follows (Page 13 Line 289-295):

As many cell line subtypes can have varying nuclear size, shape, and complexity, their N:C ratio can be variable. It is beyond the capabilities of the currently presented technique to definitively state the cell subtype. However, a preliminary comparison between the N:C ratios of malignant and non-malignant cell lines does point towards a potential use for determining whether an individual cell is oncogenically transformed.

3. Also SK-BR3 is a breast cancer cell line derived from the pleural fluid, a metastatic site. As such it may not be representative of the primary tumour but may be aneuploid.

The reviewer raises a valid point regarding the ploidy of cells in the primary tumour and those that have metastasized. However, the aim of this work was not to determine the anatomical site of origin of the cells, but to instead demonstrate the ability of IFC to assess cellular N:C ratio in a high-throughput manner. We have noted this as a limitation in the “Discussion” section of the manuscript as follows (Page 13 Line 288-295):

Moreover, our future work will consider the effects of aneuploidy in the context of the N:C ratio. As many cell line subtypes can have varying nuclear size, shape, and complexity, their N:C ratio can be variable, and it is beyond the capabilities of the currently presented technique to definitively state the anatomical origin of a single given cell a priori. However, a preliminary comparison between the N:C ratios of malignant and non-malignant cell lines does point towards a potential use for determining whether an individual cell is oncogenically transformed. Moreover, since this study focused on the presentation of a novel high-throughput technique to characterize the N:C ratio in multiple cell lines, the effects of aneuploidy were not considered.

4. The loss of >80% of cells in the analysis raises questions over the methodology, instrument set up, validity of the data and applicability in practice. Did the cell loss occur pre-analytical as a consequence of the trypsin causing cytological damage to cell membranes or is the loss due to instrument factors? Can the authors address this high cell loss? The cell edge gradient/focus and clipped cells data shows a high level of flow core instability (>60 cell edge gradient). This reviewer does not agree with the authors comment that this is a standard limitation or operation of imaging flow cytometry. This needs to be addressed.

In line with the Reviewer’s comment, we do note that we are choosing a higher gradient RMS value (>60) but have seen that the choice of this high gradient value leads to cells in-focus that enable accurate brightfield classification (47, 50, 51, 52). In addition, we have previously observed the “halo effect” during cell masking in the IDEAS software (27) and thus chosen a high gradient threshold to minimize this effect. Moreover, in line with the limitations of subjectivity of IFC analysis, we have noted in the manuscript that our gating process might be different than other users. We recognize that our approach is not a one-size fits all method, and that slight modifications to thresholds or gradient levels may be required when analyzing different cell types, or even the same cell line for different applications.

The reviewer’s concern that the trypsin could be causing cytological damage to the cell membranes has been noted (and discussed in our reply Reviewer 1’s first comment), and we have modified the manuscript to further discuss this (Page 14 Line 304-309):

In addition, we hypothesize that trypsin’s known damaging effects on the cell membrane (43) may have led to cell fragmentation that could have led to exclusion of affected cells during the post-processing of the acquired IFC images and account for the high cell loss in our IFC post-processing analysis. For example, the post-processing steps that gate single cell IFC images could have removed cell images that contained both the cell and its corresponding fragment due to trypsinization.

The future use of machine learning to assess the N:C ratio by our group would remove these gating steps to minimize cell loss during post-processing analysis and have previously noted this in the manuscript (Page 15-16 Line 328 – 338; most of this text is not highlighted or in red as it was previously included in the original manuscript):

“We have observed these common drawbacks to IFC in our previous work (47) and other groups have sought to improve the gating process (48, 49). To eliminate these disadvantages of the IFC analysis workflow, our future work will focus on the use of computer vision and machine learning strategies to assess the N:C ratio. Here, no gating strategies are implemented to limit the loss of IFC images. Our group combined computer vision and machine learning strategies in the context of red blood cell storage lesions (50, 51). We look to further incorporate techniques used by Doan et al. (52), Blasi et al. (53), and Hennig et al. (54) that use an unsupervised or weakly supervised, deep-learning method (55) for the assessment of the N:C ratio in both non-malignant and malignant cell lines. This would overcome drawbacks of IFC manual gating and user bias to provide an objective assessment of cell malignancy in a high-throughput context.

27.	Moore, MJ, Sebastian JA, Kolios MC. Determination of cell nucleus-to-cytoplasmic ratio using imaging flow cytometry and a combined ultrasound and photoacoustic technique: a comparison study. Journal of Biomedical Optics. 2019 Oct 1;24(10):1–10.

43. Revel JP, Hoch P, Ho D. Adhesion of culture cells to their substratum. Experimental Cell Research. 1974 Mar;84(1–2):207–18.

47. Pinto RN, Sebastian JA, Parsons MJ, Chang TC, Turner TR, Acker JP, et al. Label‐Free Analysis of Red Blood Cell Storage Lesions Using Imaging Flow Cytometry. Cytometry. 2019 Jul 11;cyto.a.23846.

48. The FlowCAP Consortium, The DREAM Consortium, Aghaeepour N, Finak G, Hoos H, Mosmann TR, et al. Critical assessment of automated flow cytometry data analysis techniques. Nat Methods. 2013 Mar;10(3):228–38.

49. Lo K, Brinkman RR, Gottardo R. Automated gating of flow cytometry data via robust model-based clustering. Cytometry. 2008 Apr;73A(4):321–32.

50. Doan M, Sebastian JA, Pinto RN, McQuin C, Goodman A, Wolkenhauer O, et al. Label-free assessment of red blood cell storage lesions by deep learning [Internet]. Bioinformatics; 2018 Jan [cited 2019 Sep 16]. Available from: http://biorxiv.org/lookup/doi/10.1101/256180

51. Doan M, Sebastian JA, Caicedo J, Seigert S, Roch A, Turner TR, et al. Objective assessment of stored blood quality by deep learning. Proc Natl Acad Sci USA. 2020 Forthcoming.

52. Doan M, Case M, Masic D, Hennig H, McQuin C, Caicedo J, et al. Label‐Free Leukemia Monitoring by Computer Vision. Cytometry. 2020 Apr;97(4):407–14.

53. Blasi T, Hennig H, Summers HD, Theis FJ, Cerveira J, Patterson JO, et al. Label-free cell cycle analysis for high-throughput imaging flow cytometry. Nat Commun. 2016 Apr;7(1):10256.

54. Hennig H, Rees P, Blasi T, Kamentsky L, Hung J, Dao D, et al. An open-source solution for advanced imaging flow cytometry data analysis using machine learning. Methods. 2017 Jan;112:201–10.

55. Caicedo JC, McQuin C, Goodman A, Singh S, Carpenter AE. Weakly Supervised Learning of Single-Cell Feature Embeddings. In: 2018 IEEE/CVF Conference on Computer Vision and Pattern Recognition [Internet]. Salt Lake City, UT: IEEE; 2018 [cited 2020 Aug 18]. p. 9309–18. Available from: https://ieeexplore.ieee.org/document/8579068/

5. DRAQ-5 stain as demonstrated in Figure 1 shows variable intensity and quality between the 4 cell lines. Were DRAQ-5 titrations performed on the cell lines to standardise masking and stain intensity measurements? How did the “masking” strategy take this variability into account? Is the variable intensity of DRAQ-5 stain related to the cell line, stage of cell cycle or ploidy? Were any other fluorescent nuclear stains assessed? Would alternate stain have addressed the leakage issue?

To account for the variability in the DRAQ-5 intensity across cell lines, our custom workflow in IDEAS gates each cell line individually based on their respective nuclear uptake of the DRAQ-5 dye. The same threshold is not used across all cell lines to account for this variation in nuclear uptake across cell lines. We have clarified this in the manuscript as follows (Page 7 Line 134-137):

Through gating for solely DRAQ-5-positive cells in plot IV, we exclude cell images containing calibration beads, which are required for alignment of the sample stream during imaging. The rationale for this gate is based on the visual clustering of cells we see in the scatter plot based on area and high pixel intensity.

Although extensive studies exploring the variability of nuclear staining of DRAQ-5 have not been done, Hoechst 33342 (i.e., another very common nuclear dye) has been determined empirically to be cell-type and concentration-dependent (29-31). Thus, we believe that the variation in nuclear uptake of the DRAQ-5 could also be dependent on these factors. We account for this variability through our individualized gating thresholds per cell line. In addition, these cells are four sample cells selected from thousands of cells thus it is reasonable to expect variability across the entire analyzed population. We have noted this in the revised manuscript in the “Results” section as follows (Page 8 Line 170-174):

There is noticeable variability in the nuclear uptake of the DRAQ-5 as depicted in the 2nd vertical panel of Fig 1(c-g). The variability of nuclear dyes has been studied and empirically determined as being cell cycle, cell-type, and concentration dependent (29-31). To account for this variability in dye uptake, the fluorescent intensity gating process (Fig 1.a.IV) is individualized for each cell line.

29. Gomes, C.J., Harman, M.W., Centuori, S.M. *et al.* Measuring DNA content in live cells by fluorescence microscopy. *Cell Div* **13,** 6 (2018). <https://doi.org/10.1186/s13008-018-0039-z>

30. Durand RE. Use of Hoechst 33342 for cell selection from multicell systems. J Histochem Cytochem. 1982;30:117–22. <https://doi.org/10.1177/30.2.6174559>

31. Scharenberg CW, Harkey MA, Torok-Storb B. The ABCG2 transporter is an efficient Hoechst 33342 efflux pump and is preferentially expressed by immature human hematopoietic progenitors. Blood. 2002. <https://doi.org/10.1182/blood.v99.2.507>.

In addition, other fluorescent nuclear stains were not assessed in these experiments, but we believe that the study of the effects of multiple nuclear dyes on nuclear uptake and the N:C ratio could provide the basis for subsequent studies. We hypothesize that the leakage referred to by Reviewer 1 is due to cytological damage due to the trypsin treatment, therefore we suspect that an alternate stain would not have addressed the leakage issue. We have included both these points as limitations and point for future work in the “Discussion” section as follows (Page 13 Line 284-288):

Additionally, DRAQ-5 was the only nuclear dye used in this study. The examination of the effects of various nuclear stains, their dye uptake, and effects on the N:C ratio will be the subject of future studies. However, we hypothesize that an alternate dye would not address the leakage problem as DRAQ5 has shown high nuclear localization in other studies (42).

42. Smith PJ, Blunt N, Wiltshire M, Hoy T, Teesdale-Spittle P, Craven MR, et al. Characteristics of a novel deep red/infrared fluorescent cell-permeant DNA probe, DRAQ5, in intact human cells analyzed by flow cytometry, confocal and multiphoton microscopy. Cytometry. 2000 Aug 1;40(4):280–91.

6. Figure 2f shows SK-BR-3 cells with bimodal distribution of N:C ratios. Is this variability due to stain intensity, cell proliferation or cell viability? Have any other DNA stains been used? Would these have addressed the leakage issue?

We attribute the bi-modal distribution of the N:C ratio for the SK-BR-3 cells to the cell’s proliferation and stage of the cell cycle. We addressed this in the manuscript (Page 12-13 Line 267-276; most of this text is not highlighted or in red as it was previously included in the original manuscript):

From Fig 2 (a-d), the measurements of cell diameter were consistent with the measurement of nuclear diameter over a range of cell sizes. This relationship between cell and nuclear size throughout the cell cycle is in line with what has been observed in the literature (36-38) and the future studies will examine the effect of the cell cycle (37) on the N:C ratio. In particular, the distribution of SK-BR-3 cell diameter found in Fig 2a, b does not show a Gaussian-like shape like the other cell lines did. The shape of the cell size distribution can be affected by the length of time the cell spends in each phase of the cell cycle. In this work however, the time in the S/G2/M phase is similar for all the cell lines studied (SKBR3 - 36%, HT-29 - 32%, CAKI-2 - 35%) (39-41). Therefore, it is possible that the size variability of the SK-BR-3 cell line is inherent to this cell line.

36. Jorgensen P, Edgington NP, Schneider BL, Rupes I, Tyers M, Futcher B. The size of the nucleus increases as yeast cells grow. Mol Biol Cell. 2007 Sep;18(9):3523–32.

37. Chu F-Y, Haley SC, Zidovska A. On the origin of shape fluctuations of the cell nucleus. Proc Natl Acad Sci USA. 2017 Sep 26;114(39):10338–43.

38. Swanson JA, Lee M, Knapp PE. Cellular dimensions affecting the nucleocytoplasmic volume ratio. J Cell Biol. 1991 Nov;115(4):941–8.

39. Zou C-F, Jia L, Jin H, Yao M, Zhao N, Huan J, et al. Re-expression of ARHI (DIRAS3) induces autophagy in breast cancer cells and enhances the inhibitory effect of paclitaxel. BMC Cancer. 2011 Dec;11(1):22.

40. Tsuji PA, Naranjo-Suarez S, Carlson BA, Tobe R, Yoo M-H, Davis CD. Deficiency in the 15 kDa Selenoprotein Inhibits Human Colon Cancer Cell Growth. Nutrients. 2011 Sep 5;3(9):805-17.

41. Liu Y, Qian J, Li X, Chen W, Xu A, Zhao K, et al. Long noncoding RNA BX357664 regulates cell proliferation and epithelial-to-mesenchymal transition via inhibition of TGF-&#x3B2;1/p38/HSP27 signaling in renal cell carcinoma. Oncotarget [Internet]. 2016 Dec 6 [cited 2020 Apr 29];7(49). Available from: http://www.oncotarget.com/fulltext/12937

7. How has nuclear complexity been taken into consideration when the nuclear diameter was measured?

We are unsure what the Reviewer defines as nuclear complexity, but we will assume in this answer that they are referring to the shape of the nucleus. Our technique includes cells that have both circular and non-circular nuclei. Similar to our reply to comment #9, the Diameter feature provides the diameter of a circle that has the same area of the masked object. Thus, it is a limitation of our measurement technique that is addressed in the “Discussion” section of the revised manuscript as follows (Page 15 Line 323-326):

Similarly, the use of the “Diameter” feature to mask the cell or nucleus provides the diameter of a circle that has the same area as the masked cell or nucleus (28). This step introduces the possibility of overestimating the nuclear diameter in cases of nuclear shape variability.

28. Amnis. IDEAS® ImageStream Analysis Software User’s Manual. Version 2015;6:2.

8. The cell cycle and nuclear content was acknowledged as influencing the N:C results. How did different stages of cell cycle affect nuclear measurements and subsequent N:C ratio?

We previously mentioned in the manuscript (Page 13 Line 272-274) that “the shape of the cell size distribution can be affected by the length of time the cell spends in each phase of the cell cycle. In this work however, the time in the S/G2/M phase is similar for all the cell lines studied (SKBR3 - 36%, HT-29 - 32%, CAKI-2 - 35%) (39-41).” We do not mention the effect of the cell cycle on the nuclear size. Since it is established that the nucleus increases in size during the cell cycle (37), it is possible that the N:C ratio could have been overestimated due to this nuclear growth. We have now added to the manuscript (Page 12-13 Line 268-270):

This relationship between cell and nuclear size throughout the cell cycle is in line with what has been observed in the literature (36-38) and the future studies will examine the effect of the cell cycle (37) on the N:C ratio.

36. Jorgensen P, Edgington NP, Schneider BL, Rupes I, Tyers M, Futcher B. The size of the nucleus increases as yeast cells grow. Mol Biol Cell. 2007 Sep;18(9):3523–32.

37. Chu F-Y, Haley SC, Zidovska A. On the origin of shape fluctuations of the cell nucleus. Proc Natl Acad Sci USA. 2017 Sep 26;114(39):10338–43.

38. Swanson JA, Lee M, Knapp PE. Cellular dimensions affecting the nucleocytoplasmic volume ratio. J Cell Biol. 1991 Nov;115(4):941–8.

39. Zou C-F, Jia L, Jin H, Yao M, Zhao N, Huan J, et al. Re-expression of ARHI (DIRAS3) induces autophagy in breast cancer cells and enhances the inhibitory effect of paclitaxel. BMC Cancer. 2011 Dec;11(1):22.

40. Tsuji PA, Naranjo-Suarez S, Carlson BA, Tobe R, Yoo M-H, Davis CD. Deficiency in the 15 kDa Selenoprotein Inhibits Human Colon Cancer Cell Growth. Nutrients. 2011 Sep 5;3(9):805-17.

41. Liu Y, Qian J, Li X, Chen W, Xu A, Zhao K, et al. Long noncoding RNA BX357664 regulates cell proliferation and epithelial-to-mesenchymal transition via inhibition of TGF-&#x3B2;1/p38/HSP27 signaling in renal cell carcinoma. Oncotarget [Internet]. 2016 Dec 6 [cited 2020 Apr 29];7(49). Available from: http://www.oncotarget.com/fulltext/12937

9. The nuclear masks/fluorescent images shown in Figure 1B-E for the four cell lines highlight the potential for variability in nuclear content/configuration. The calculation of nuclear and cytoplasmic area, and by extension the N:C ratio is based on the “diameter” feature of the image masks for the nucleus and cell – calculations with “diameter” is only mathematically consistent if the objects have high circularity. Can the authors clarify the circularity feature/masks in the IDEAS software?

We have revised the manuscript to note this comment by Reviewer 2 in the “Methods” section (Page 7, Line 139-141) as follows:

Here, the eroded masks were used to determine the cell diameter where IDEAS provided the diameter of a circle that has the same area as the eroded masks (28).

28. Amnis. IDEAS® ImageStream Analysis Software User’s Manual. Version 2015;6:2.

Reviewer #2:

1. The authors largely misunderstand the cited references 9-11. While enlarged N/C ratio is one morphologic feature of malignancy, in general, that is seen in both histology and cytology, the N/C ratio is most easily determined in cytologic specimens and not histology, and is currently mostly used in the study of urinary cytology. While the authors correctly cite the references 9-11, they incorrectly say on page 3 that this is the examination of histologic sections. In fact, this is not true, as cytology specimens are not sectioned and are not referred to as "histology" - the slides are fixed to a glass slide using any number of methods (primarily alcohol-based fixation or air fixation). Therefore, while the entire 3D structure of the cell can be examined by adjusting the microscope focus, the N/C ratio is typically calculated in one “Z-plane” in which both the nuclear contour and cytoplasmic contour appear most in focus. The authors should adjust their text to take this into account, and it would be relevant to comment on how the flow cytometric method determines which “Z-plane” contains the calculated N and C diameter values.

In reviewing references 9-11 and Reviewer 2’s comment, we have modified the text by removing our previous claims on Page 3 Line 59-62 to now state:

Although histology and cytology are the gold standards of the N:C ratio assessment method, many studies have reported the interobserver variability that exists in during visual quantitation (9-11). In practice, cytology, where slides of biological specimen are fixed to a glass slides and examined, is another method used to assess the N:C ratio.

To address Reviewer 2’s second comment, we want to highlight that the ImageStream analyzes cells in suspension that have been hydrodynamically focused in flow and illuminated by a brightfield laser source. One of the trade-offs of using an IFC system is that to achieve high-throughput measurements the cells are imaged in a flow stream that traverses a fixed microscope focus. Thus, there is no optimal “Z-plane” selected on an individual cell basis when calculating the nucleus and cell diameter.

9. Layfield LJ, Esebua M, Frazier SR, Hammer RD, Bivin WW, Nguyen V, et al. Accuracy and Reproducibility of Nuclear/Cytoplasmic Ratio Assessments in Urinary Cytology Specimens: REPRODUCIBILITY OF NUCLEAR/CYTOPLASMIC RATIO ESTIMATES. Diagn Cytopathol. 2017 Feb;45(2):107–12.

10. Zhang ML, Guo AX, VandenBussche CJ. Morphologists overestimate the nuclear-to-cytoplasmic ratio: Overestimation of N:C Ratio. Cancer Cytopathology. 2016 Sep;124(9):669–77.

11. Vaickus LJ, Tambouret RH. Young investigator challenge: The accuracy of the nuclear-to-cytoplasmic ratio estimation among trained morphologists: Accuracy of N:C Ratio Estimation. Cancer Cytopathology. 2015 Sep;123(9):524–30.

2. The authors use only malignant cell lines and it would have been nice to have used non-malignant control cells as well, even if not clonally expanded.

Our revised manuscript now incudes N:C data using the MCF10A cell line (a known normal breast cell model derived from non-malignant breast tissue) that demonstrates a clear difference between the N:C ratio of normal and malignant cells. We kindly ask the Reviewer to refer to Reviewer 1’s first comment and our content and figures there.

3. The authors should note that most epithelial malignancies taken directly from a patient will have cohesive cells and tissue fragments. To use this method would require breaking the cells apart, which may alter the cellular natural state, or would be biased towards individual cells, which typically are more pleomorphic than cells that remain in fragments. However, the method is immediately useful in malignancies which tend to be single cells (e.g. urothelial carcinoma and lymphoma/leukemia).

We have added to the “Discussion” section of the manuscript to note this comment by Reviewer 2 as follows (Page 10-11 Line 214 – 224):

Currently, histology is the gold standard assessment method for the determination of the N:C ratio but it cannot be practically used when analyzing large populations of cells. Histology is advantageous when examining cohesive cells and tissue fragments, but it cannot be optimally used when analyzing large populations of cells. Techniques which require a single cell suspension, such as IFC, would require special preparation techniques if using samples obtained from punch biopsies. These methods could in turn alter the cellular morphology and state of the cells and impact the resultant analysis. However, in many high-throughput clinical contexts, such as bodily fluid analysis for the detection of circulating tumor cells, minimal residual disease and hematological diseases, cytology would be cumbersome. Thus, an objective high-throughput technique to assess the N:C ratio would provide an approach that would provide this measurement more reliably and with larger sample datasets.

4. The authors should mention that N/C ratio is an important cytomorphologic feature of malignancy for many, but not all, tissue types. For instance, melanoma tends to have more cytoplasm and lower N/C ratios than urothelial carcinoma. Carcinomas can have greatly varied N/C ratios. However, it is true that a very high N/C ratio (>0.7) is usually not seen in benign cells.

We have revised the manuscript to note this in the “Introduction” of the manuscript (Page 3 Line 56-65):

Clinicians have identified an enlarged nucleus as one of the most prevalent characteristics of several types of malignant cells (5-7). The enlarged nucleus of malignant cells led to the development of the nucleus-to-cytoplasmic (N:C) ratio, defined as the ratio of the cross-sectional area of the nucleus divided by that of the cytoplasm (8)…In addition, although this metric is used in many tissue types (e.g., urothelial carcinoma), in others (e.g., melanoma), lower N:C ratios (12) despite malignancy prevent adoption of the N:C ratio as a staging method.

12. Creager AJ, Pitman MB, Geisinger KR. Cytologic features of clear cell sarcoma (malignant melanoma) of soft parts: a study of fine-needle aspirates and exfoliative specimens. Am J Clin Pathol. 2002 Feb;117(2):217–24.
