# Supplementary material for: An image-based flow cytometric approach to the assessment of the nucleus-to-cytoplasm ratio
Source: PLoS One. 2021 Jun 24;16(6):e0253439. doi: 10.1371/journal.pone.0253439 (PMC8224973; doi:10.1371/journal.pone.0253439)
Supplement: Attachment — Submitted filename: Sebastian_et_al_Rebuttals_2_SubmissionCopy.docx [file pone.0253439.s003.docx]

**Sebastian et al. PLOS ONE Reviewer Rebuttals Second Revision**

We thank the editors and reviewers for thoughtfully reading our study and providing feedback, especially during these stressful times. The references and reference numbering provided in the replies to each Reviewer comment corresponds to a reference from the re-submitted manuscript.

************************************

Reviewer #2:

1. I think the authors have satisfactorily answered the criticisms of the reviewers. Given their findings compared to now included normal cell control group, I think it is remarkable how closely the N/C cut-offs correlate with the findings in two studies of urothelial carcinoma in which image-based morphometric analysis was used. While I do not think the authors necessarily need to further modify their manuscript, the findings do, in general, support the authors' findings. And, if the authors wish to pursue this area further, discussion in these manuscripts may provide valuable:
1. Digital image analysis supports a nuclear-to-cytoplasmic ratio cutoff value of 0.5 for atypical urothelial cells. PMID: 28581671 DOI: 10.1002/cncy.21883
2. Digital image analysis supports a nuclear-to-cytoplasmic ratio cutoff value below 0.7 for positive for high-grade urothelial carcinoma and suspicious for high-grade urothelial carcinoma in urine cytology specimens. PMID: 30395388 DOI: 10.1002/cncy.22061

We thank Reviewer 2 for their comments and included references. We have included them in the manuscript in the Discussion (Line 284-286): “Our N:C results are consistent with international standards of cytopathology (such as Zhang et al.’s (10) finding of an N:C ratio ≥0.7 as malignant, Hang et al.’s (42) finding of an N:C ratio cutoff value of 0.5 for atypical urothelial cells, and McIntire et al.’s (43) finding of a N:C ratio cutoff value below 0.7 for high-grade urothelial carcinoma).”

Reviewer #3:

1. The Authors describe the application of IFC to the measurement of the N:C ratio in one benign and four malignant human cell lines. The methods and their description, as well as the overall manuscript style and language, are irreproachable. However, my impression is that the study suffers from the absence of a pathologist/physician among the Authors (both in the overall design and in the manuscript itself).

Let us consider the main ideas of the study separately:

1. IFC has potential as a technique to measure N:C

The Authors convincingly show that IFC can be used to measure the N:C ratio of a population of cells. This idea is original and supported by the study design and data. However, the practical applications of this are arguable because histology (especially if empowered by computer-aided image analysis) might be better, considering that it is already routinely performed on all human cancers and does not require other tools (like an IFC).

We agree with the reviewer that histology is the most established method for cancer grading. With this work, it is not our intent to suggest that IFC should replace histology as a cancer grading evaluation technique. The goal of the present manuscript was to detail the use of IFC for the extraction and quantification of the N:C ratio of cells in suspension in a **high-throughput** context. We have modified the manuscript to reflect this reviewer’s concern:

Abstract (Lines 40-43): “This study demonstrates the use of IFC to assess the N:C ratio of cancerous and non-cancerous cells, and the promise of its use in clinically relevant high-throughput detection scenarios to supplement current workflows used for cancer cell grading.”

Discussion (Lines 250-254): The subjectivity and low-throughput nature of histology is improving using digital pathology and computer-aided image analysis; however, IFC provides an alternative for high throughput analysis with large sample analysis populations measured in a short period of time and would be useful in high-throughput clinical contexts to assess the N:C ratio of certain types of cells.

2. 2. the N:C ratio (measured by IFC) can be used as a discriminator of malignancy

In my opinion, this premise is flawed. It will be discussed below.

--------

56: "Clinicians have identified an enlarged nucleus as one of the most prevalent characteristics of malignant cells"

An enlarged nucleus is definitely a characteristic of some neoplasms. However, it is neither sensitive nor specific. There are benign cells with huge nuclei (think symplastic leiomyoma) and malignant cells with tiny nuclei (signet ring cell cancers, foamy prostate cancer). Similarly, the N:C ratio is increased in most cancers, but again it is neither sensitive nor specific. There are benign cells with alarmingly high N:C ratios (lymphocytes) and malignant cells with very low N:C ratios (again, signet ring cells). Furthermore, some neoplasms are by definition cytologically indistinguishable from their benign counterparts (and thus they share an identical N:C ratio). This is the case of follicular thyroid adenoma/carcinoma and several other endocrine neoplasms.

We appreciate the Reviewers comments. In our initial manuscript, we did note this lack of specificity and sensitivity in the Introduction Lines 64-66:

“…although this metric is used in many tissue types (e.g., urothelial carcinoma), in others (e.g., melanoma), lower N:C ratios (12) despite malignancy and higher N:C ratios in normal cells (e.g., lymphocytes) prevent adoption of the N:C ratio as a grading method.”

Based on Reviewer 3’s comments, we have modified the text to reflect that the N:C ratio is not a universal metric discriminator of malignancy but rather a metric that can be used in some cases (e.g., urothelial carcinoma [1-3]). This was done in the:

Abstract (Lines 40-43): “This study demonstrates the use of IFC to assess the N:C ratio of cancerous and non-cancerous cells, and the promise of its use in clinically relevant high-throughput detection scenarios to supplement current workflows used for cancer cell grading.”

Introduction (Lines 57-60): “Clinicians have identified an enlarged nucleus as a prevalent characteristic of certain types of malignant cells (5-7). The enlarged nucleus of these malignant cells led to the development of the nucleus-to-cytoplasmic (N:C) ratio, defined as the ratio of the cross-sectional area of the nucleus divided by that of the cytoplasm (8).”

Discussion (Lines 212-214): “The N:C ratio can be used as a histological metric in grading malignant disease in certain tissue types. In these tissues, an enlarged nucleus has become a hallmark due to the abundance of chromatin present within malignant cells (1).”

Discussion (Lines 256-258): “The lack of high throughput techniques available to analyze the N:C ratio provides a niche opportunity for this emerging cytometric method to be used in certain cell types.”

1. Layfield LJ, Esebua M, Frazier SR, Hammer RD, Bivin WW, Nguyen V, et al. Accuracy and Reproducibility of Nuclear/Cytoplasmic Ratio Assessments in Urinary Cytology Specimens: REPRODUCIBILITY OF NUCLEAR/CYTOPLASMIC RATIO ESTIMATES. Diagn Cytopathol. 2017 Feb;45(2):107–12.
2. Hang J-F, Charu V, Zhang ML, VandenBussche CJ. Digital image analysis supports a nuclear-to-cytoplasmic ratio cutoff value of 0.5 for atypical urothelial cells. Cancer Cytopathol. 2017 Sep;125(9):710–6.
3. McIntire PJ, Snow JT, Elsoukkary SS, Soong L, Sweeney J, Robinson BD, et al. Digital image analysis supports a nuclear-to-cytoplasmic ratio cutoff value below 0.7 for positive for high-grade urothelial carcinoma and suspicious for high-grade urothelial carcinoma in urine cytology specimens. Cancer Cytopathol. 2019 Feb;127(2):120–4.
3. 211: "The N:C ratio is widely used as a histological metric in staging malignant disease in most tissue types". This is not true, as far as I'm aware. Histological grading (not staging) of cancers depends on the histotype, but only rarely does the N:C ratio make an appearance in grading criteria. For example, prostate cancer is graded based on architecture alone; endometrial, colorectal, and lung cancers are graded mostly based on architecture; even in neoplasms in which the N:C ratio is somehow considered in grading (urothelium, breast), it is just one of several criteria.

We thank the Reviewer for their comment. We have modified the text to reflect their comments in the:

Abstract (Lines 25-26): The nucleus-to-cytoplasm ratio (N:C) can be used as one metric in histology for grading certain types of tumor cells.

Introduction (Lines 64-66): In addition, although this metric is used in many tissue types (e.g., urothelial carcinoma), in others (e.g., melanoma), lower N:C ratios (12) despite malignancy and higher N:C ratios in normal cells (e.g., lymphocytes) prevent adoption of the N:C ratio as a grading method.

Discussion (Lines 212-214): The N:C ratio can be used as a histological metric in grading malignant disease in certain tissue types. In these tissues, an enlarged nucleus has become a hallmark due to the abundance of chromatin present within malignant cells (1).

4. 212: "An enlarged nucleus has become a hallmark in tumor staging and grading due to an abundance of chromatin present within malignant cells". Staging and grading are two distinct things. Grading has been discussed previously. Staging of most if not all human cancers uses the AJCC TNM system which does not take the N:C ratio into account at all.

As per our reply to Reviewer 3 Comment 3, we have modified the manuscript text to reflect that the N:C ratio can be used as **one** histological metric in tumor **grading** in the Abstract, Introduction, and Discussion.

5. - 48: the Authors seem to suggest that histology as the gold standard technique to diagnose malignancy should be replaced by other, high-throughput, techniques. This is arguable, but not supported by the rest of the paper.

It was not our intent to suggest this in the manuscript. We aimed to suggest that IFC would be advantageous over histology in high-throughput clinical contexts to assess the N:C ratio of certain types of cells. Please see our reply to Reviewer 3 Comment 1 for in-text examples of our claims in the Abstract, Introduction, and Discussion.

6. - 240: what's TPM?

TPM was used as an abbreviation to two-photon microscopy and is now included in the Discussion (Line 241).

7. - 250: histological sectioning is indeed tedious, but it is performed by routine on all human cancers, so the added cost would be minimal.

If the Reviewer is referring to the monetary cost of histology, we do not suggest that IFC is inexpensive over histology in the manuscript. However, if the Reviewer is referring to the effort involved in histological sectioning, we are suggesting that conventional histological sectioning and manual evaluation of the N:C ratio via microscopy would be tedious if attempting to achieve an analysis sample size comparable to that of IFC (tens of thousands of cells).

8. - Is the N:C ratio calculated as a ratio of diameters, areas or volumes?

In our study, we calculate the N:C ratio as a ratio between the nuclear diameter and the cell diameter. It can be calculated as a ratio of either diameters, areas or volumes depending on the imaging outputs of the measurement technique [1, 2].

1. Nandakumar V, Kelbauskas L, Hernandez KF, Lintecum KM, Senechal P, Bussey KJ, et al. Isotropic 3D Nuclear Morphometry of Normal, Fibrocystic and Malignant Breast Epithelial Cells Reveals New Structural Alterations. Najbauer J, editor. PLoS ONE. 2012 Jan 5;7(1):e29230.
2. Huang H-C, Chiang S-J, Wen S-H, Lee P-J, Chen H-W, Chen Y-F, et al. Comparison of two- and three-dimensional nuclear to cytoplasm ratios in analyzing lung cancer cell lines. In: Farkas DL, Leary JF, Tarnok A, editors. Imaging, Manipulation, and Analysis of Biomolecules, Cells, and Tissues XVII [Internet]. San Francisco, United States: SPIE; 2019 [cited 2020 Apr 8]. p. 27. Available from: https://www.spiedigitallibrary.org/conference-proceedings-of-spie/10881/2507400/Comparison-of-two--and-three-dimensional-nuclear-to-cytoplasm/10.1117/12.2507400.full
9. - 59: Measuring the N:C ratio by visual approximation on histological images is indeed error-prone and operator-dependent. However, computer-aided image analysis can overcome this problem.

We thank the Reviewer for this comment. We added the following sentences to the manuscript to note the impact of computer-aided image analysis in histological analysis:

Discussion (Lines 250-254): The subjectivity and low-throughput nature of histology is improving using digital pathology and computer-aided image analysis; however, IFC provides an alternative for high throughput analysis with large sample analysis populations measured in a short period of time and would be useful in high-throughput clinical contexts to assess the N:C ratio of certain types of cells.

10. - 214: "Currently, histology is the gold standard assessment method for the determination of the N:C ratio but it cannot be practically used when analyzing large populations of cells". Partly true: digital pathology and computer-aided image analysis are overcoming this problem. The Authors themselves disprove this statement at line 225.

In Line 225, we claim that histology has been improved through digital pathology and computer-aided image analysis, in agreement with Reviewer 3. We suggest in the proceeding paragraph that although the sample populations analyzed in histology have increased because of these technologies, IFC provides an alternative that can analyze much large populations of cells in a short amount of time. Similar to other replies to this Reviewer, we are not claiming that IFC should replace histology. We are suggesting that IFC is a technology that has advantages over other modalities in certain high-throughput clinical contexts. After discussing previously published studies that use these technologies to improve histology, we have modified the Discussion (Lines 250-254) to state:

The subjectivity and low-throughput nature of histology is improving using digital pathology and computer-aided image analysis; however, IFC provides an alternative for high throughput analysis with large sample analysis populations measured in a short period of time and would be useful in high-throughput clinical contexts to assess the N:C ratio of certain types of cells.

In conclusion, IFC is an interesting and promising technique, and it can surely be used to measure the N:C ratio of cells (as this manuscript shows). However, the idea of diagnosing malignancy based on the N:C ratio (measured by IFC or otherwise) seems flawed. The study would be better reorganized by focusing on the N:C measurement by IFC as the main finding and discarding any implications on the diagnosis of malignancy.

We are grateful for this Reviewer’s comment and have modified all claims in the manuscript which imply that IFC would be able to diagnose the malignancy of a cell based solely on the N:C ratio. We refer the Reviewer to our replies to Comments 2 & 3 for specific actions which address this concern.
