# Supplementary material for: An image-based flow cytometric approach to the assessment of the nucleus-to-cytoplasm ratio
Source: PLoS One. 2021 Jun 24;16(6):e0253439. doi: 10.1371/journal.pone.0253439 (PMC8224973; doi:10.1371/journal.pone.0253439)
Supplement: Attachment — Submitted filename: Sebastian_et_al_Rebuttals_3_SubmissionCopy.docx [file pone.0253439.s004.docx]

**Sebastian et al. PLOS ONE Reviewer Rebuttals Third Revision**

We thank the editors and reviewers for thoughtfully reading our study and providing feedback, especially during these stressful times. The references and reference numbering provided in the replies to each Reviewer comment corresponds to a reference from the re-submitted manuscript.

************************************

Reviewer #2:

1. The authors have appropriately addressed all comments. However, I want to make a few corrections in their response and modifications.

For the new statement “Our N:C results are consistent with international standards of cytopathology (such as Zhang et al.’s (10) finding of an N:C ratio ≥0.7 as malignant, Hang et al.’s (42) finding of an N:C ratio cutoff value of 0.5 for atypical urothelial cells, and McIntire et al.’s (43) finding of a N:C ratio cutoff value below 0.7 for high-grade urothelial carcinoma).” please remove reference 10 here. Reference 10 is correctly cited earlier in the manuscript as it studied interobserver variability, but it did not study the impact of N/C ratio on determining whether cells were malignant or not.

We thank Reviewer 2 for their comments. We have removed Reference 10 from the mentioned sentence in the Discussion (this removal can be seen in the marked up copy of the manuscript in the Discussion Line 284).

2. Regarding the comments of reviewer #3, I agree that the N/C ratio is not in general a standard used in histopathologic examination for grading malignancies. It is true that it plays a role in some types of malignancy as increased N/C ratio is one of many features used to assess a cell for cytologic atypia. I bring this up because the authors seem to focus mostly on N/C ratio in tissue sections when the assessment is more readily done in cytologic specimens, and in cytology the only formal system that requires an N/C ratio assessment is The Paris System for urine specimens. So, while the N/C ratio is one of many cytologic features that can help diagnose malignancy or grade/differentiation of a malignancy, it depends on the specimen type and the cancer type. I would recommend double checking the manuscript to ensure the introduction and discussion is consistent with this idea. In part, because the N/C ratio can be difficult to assess in both histology and cytology, is is important that the authors have shown a way to objectively measure the N/C ratio and correlate ratios with malignant vs. benign cells. This can help show the degree to which N/C ratio variations are associated with malignancy, at least in certain cell types, even if no single feature can be used, in most cases, to determine whether a cell is malignant or benign.

We thank Reviewer 2 for their comments. In response to Reviewer 3 in our second rebuttal document, we modified multiple phrases in the Abstract, Introduction, and Discussion to clarify that while the N/C ratio is one of many cytologic features that can help diagnose malignancy or grade, it depends on the specimen and cancer type. We would like to draw the Reviewer’s attention to the following sections:

Abstract (Lines 25-26): The nucleus-to-cytoplasm ratio (N:C) can be used as one metric in histology for grading certain types of tumor cells.

Abstract (Lines 40-43): “This study demonstrates the use of IFC to assess the N:C ratio of cancerous and non-cancerous cells, and the promise of its use in clinically relevant high-throughput detection scenarios to supplement current workflows used for cancer cell grading.”

Introduction (Lines 57-60): “Clinicians have identified an enlarged nucleus as a prevalent characteristic of certain types of malignant cells (5-7). The enlarged nucleus of these malignant cells led to the development of the nucleus-to-cytoplasmic (N:C) ratio, defined as the ratio of the cross-sectional area of the nucleus divided by that of the cytoplasm (8).”

Introduction (Lines 64-66): In addition, although this metric is used in many tissue types (e.g., urothelial carcinoma), in others (e.g., melanoma), lower N:C ratios (12) despite malignancy and higher N:C ratios in normal cells (e.g., lymphocytes) prevent adoption of the N:C ratio as a grading method.

Discussion (Lines 212-214): The N:C ratio can be used as a histological metric in grading malignant disease in certain tissue types and cytologic specimens. In these biological samples, an enlarged nucleus has become a hallmark due to the abundance of chromatin present within malignant cells (1).

Discussion (Lines 256-258): “The lack of high throughput techniques available to analyze the N:C ratio provides a niche opportunity for this emerging cytometric method to be used in certain cell types.”

Reviewer #3:

1. All comments have been addressed in a satisfactory manner. State-of-the-art high-throughput whole slide scanners coupled with appropriate software are already usable to get N:C data in high-throughput contexts; however I have to agree that until these become ordinary, they represent an extra cost (not unlike an IFC).

We thank Reviewer 3 for their insight and comments.
